# Population Pharmacokinetics of Vancomycin in Kidney Transplant Recipients: Model Building and Parameter Optimization

**DOI:** 10.3389/fphar.2020.563967

**Published:** 2020-10-06

**Authors:** Kui-fen Ma, Yi-xi Liu, Zheng Jiao, Jun-hao Lv, Ping Yang, Jian-yong Wu, Si Yang

**Affiliations:** ^1^Department of Pharmacy, The First Affiliated Hospital, Zhejiang University School of Medicine, Hangzhou, China; ^2^Department of Pharmacy, Shanghai Chest Hospital, Shanghai Jiao Tong University, Shanghai, China

**Keywords:** vancomycin, therapeutic drug monitoring, population pharmacokinetics, kidney, transplantation vancomycin, transplantation Key Points

## Abstract

**Background:**

Depending on the renal function of patients and many other influencing factors, studies on vancomycin pharmacokinetics show significant inter- and intra-individual variability. The present study was conducted using a population pharmacokinetics method to investigate the pharmacokinetic parameters and identified their influencing covariates for intravenous vancomycin in adult kidney transplant recipients.

**Methods:**

The drug monitoring data included 56 adult renal transplant recipients who received intravenous vancomycin as prophylactic medication. The analysis was performed by a population approach with NONMEM. Data were collected mainly during the first week after transplantation. Monitoring of vancomycin trough concentration in blood was initiated mainly 3–5 days after the initial administration.

**Results:**

The one-compartment open model was optimal and adequately described the data. Body weight (WT) and estimated glomerular filtration rate (GFR) were identified as significant covariates of the pharmacokinetic parameters CL and V of intravenous vancomycin in the kidney transplant patients. The typical values of vancomycin CL and V were 2.08 L h^-1^ and 63.2 L, respectively. A dosage strategy scheme according to model results was also designed.

**Conclusion:**

Both WT and GFR of the kidney transplant patients positively influence the pharmacokinetic parameters CL and V for intravenous vancomycin. Our population pharmacokinetic model provides a reference for vancomycin dosage adjustment in kidney transplant recipients.

## Highlights

Population pharmacokinetic analysis of vancomycin in the kidney transplant recipients revealed that body weight (WT) and estimated glomerular filtration rate (GFR) of patients were significant covariates of pharmacokinetic parameters CL and V. WT and GFR both positively influence CL and V of vancomycin after intravenous administration. In kidney transplant patients, WT and GFR should be considered for vancomycin dosage according to recommended schemes, and blood concentration of vancomycin should be monitored for the entire duration to avoid insufficient exposure-induced ineffective treatment and bacterial resistance, and drug overdose-induced toxicities.

## Introduction

Vancomycin is a narrow-spectrum glycopeptide antibiotic derived from *Streptomyces orientalis* strain and is mainly used for infectious diseases caused by most gram-positive bacteria. Currently, vancomycin is widely used as an antimicrobial agent in surgical operation and organ transplant patients (Siebers et al., 2018; Imlay et al., 2019) and is also used as a preventive and therapeutic drug in the kidney transplant patients (Splinter et al., 2018). Therapeutic drug monitoring (TDM) and assessment of multiple factors affecting its pharmacokinetics and efficacy should be considered in clinical use.

The minimum inhibitory concentration (MIC) of vancomycin for most sensitive bacteria is 0.1–2 μg ml^-1^ (Bhongsatiern et al., 2015; Bruniera et al., 2015). Many of the adverse reactions of vancomycin such as bone marrow hematopoietic toxicity and nephrotoxicity are closely related to the blood concentration and area under the curve (AUC) of vancomycin (Bruniera et al., 2015; Gyamlani et al., 2019). Pharmacokinetics, blood concentration, efficacy, and toxicity of vancomycin vary according to various factors including patient’s physiology, pathology, and combined medication (Giuliano et al., 2010; Chu et al., 2016; Bakke et al., 2017; Hu et al., 2018). For reducing adverse reactions and low dose-induced drug resistance, the blood peak concentration (C_max_) of vancomycin should be kept between 20–40 μg ml^-1^ in clinical application (Martin et al., 2010; Steinmetz et al., 2015). Blood drug concentration should be monitored during vancomycin therapy, especially for patients with renal dysfunction, newborns, children, and elderly patients, and patients with combined use of other drugs with adverse reactions to the kidney (Hahn et al., 2015; Arfa et al., 2016; Chu et al., 2016). Vancomycin exposure is commonly represented by C_min_ or AUC, and C_min_ can also be used as a predictor of daily AUC (AUC_0-24h_). As the AUC_0-24h_/MIC for therapeutic efficacy is suggested to be≥400 mg·h L^-1^, a C_min_ value of 11 μg ml^-1^ can be used as an optimal predictor of AUC_0-24h_ for suitable vancomycin exposure (Bel Kamel et al., 2017).

For time-dependent antimicrobial drug vancomycin, C_min_ has a direct and decisive effect on its efficacy and low dose-induced resistance, and is the most commonly used indicator for TDM and dosage adjustment (Avent et al., 2013; Ye et al., 2013). The TDM of vancomycin C_min_ should be initiated after concentration has achieved a steady state in the blood, usually before the fifth dose, and blood samples must be taken 30 min before administration of the drug (Ye et al., 2014; Cardile et al., 2015). In recent years, all the international guidelines and consensuses have recommended that vancomycin C_min_ should be kept between 15–20 μg ml^-1^ for complex and severe infections (Matsumoto et al., 2013; Ye et al., 2016).

Studies have found that pharmacokinetic parameters of vancomycin are associated with the renal function, age, weight, combined medications, and other factors (Chu et al., 2016; Covvey et al., 2019; Yahav et al., 2019). Based on the population pharmacokinetic approach, we can screen out variables that influence the parameters of vancomycin pharmacokinetics, and establish formulas describing individual pharmacokinetic parameters. With the parameter-describing formulas, it is convenient to calculate and adjust individual dosages of vancomycin, which can improve the attainment of target concentrations and reduce the occurrence of adverse reactions and bacterial resistance (Guilhaumou et al., 2016; Monteiro et al., 2018). The kidney transplant patients have limited compensatory ability, and thus have decreased clearance rates of vancomycin and other nephrotoxic drugs, which may induce drug accumulation and toxicity to the transplanted kidney. Therefore, establishment of vancomycin population pharmacokinetic models in the kidney transplant patients becomes priority for clinical pharmacists to develop individualized medication. Although many studies have reported that the pharmacokinetics of vancomycin is altered among different subpopulations, little is known about vancomycin pharmacokinetics in the kidney transplant recipients.

This study used TDM data to establish population pharmacokinetic models of vancomycin in renal transplant recipients to assist in the design of individualized dosage regimens of vancomycin. Based on the pharmacokinetic parameters and the significant covariates identified from the models, the clinical dosages can be accurately designed or adjusted to ensure that the vancomycin exposure level is within the effective therapeutic range, thereby reducing the occurrence of severe adverse reactions.

## Materials and Methods

### Patients and Data Collection

We conducted a retrospective collection and analysis of data from 56 adult recipients who received vancomycin as prophylactic medication following kidney transplant operation in a single center. All these patients adopted routine therapeutic drug monitoring. The transplant surgeries were performed between March 8 and June 21 in 2017. Data were collected from the beginning of vancomycin administration to the time after transplantation. The following data or covariates were recorded and collected: (a) Basal characteristics: number of qualified recipients (male/female), ages, body weight (WT), dialysis duration pretransplantation; (b) Laboratory examination reports: aspartate aminotransferase (AST), alanine aminotransferase (ALT), proteinemia (PROT), hemoglobin, and serum creatinine concentration (sCr); (c) Parameters related to surgery: kidney donors, duration of cold ischemia; (d) Medical care: durations of hospitalization and vancomycin treatment; and (e) Usage of immunosuppresive drugs: prednisone, mycophenolate mofetil, cyclosporine, and tacrolimus. The glomerular filtration rate (GFR) was estimated from the sCr according to the published formula [GFR (ml min^-1^): GFR = 2.104*sCr(μM) - 1.154*age - 1.154*(0.742 for female)*1.233(correction for Chinese)] (Chu et al., 2020).

### Drug Administration

Intravenous vancomycin was administered as a prophylactic medication for the transplant patients at a dosage of 500 mg per administration. On the initial day of postoperative period, only one dose of vancomycin (500 mg) was administered. In the following days, vancomycin was administered intravenously 1–4 times each day (500–2,000 mg), depending on the clinical evidence of efficacy and toxicity and the trough plasma concentrations that should be maintained at relatively suitable levels. Other concomitantly used drugs, such as prednisone, mycophenolate mofetil, cyclosporine, and tacrolimus were administered orally or intravenously once or twice a day, and their usages and doses were based on clinical necessity and safety.

### Therapeutic Drug Monitoring

For the monitoring of vancomycin C_min_, venous blood samples were initially collected mainly 3–5 days after the first administration (after 4–5 doses of intravenous vancomycin) following transplantation. In the subsequent periods, blood samples for TDM were collected 2–5 times about 30 min before the morning dose of vancomycin, until the detected concentrations stabilized. The venous blood was centrifuged at 1,000 g for 4 min and a volume of 100 ml supernatant plasma was mixed with 100 ml of 5% perchloric acid solution. The mixture was fully vortex blended and then centrifuged at 14,000 g for 5 min. A 20 µl aliquot of the supernatant was sampled and separated, and the plasma concentration of vancomycin was determined by high-performance liquid chromatography (HPLC, Agilent 1260). AHC-C_18_ column (250 mm × 4.6 mm, 5 μm; ShimadzuCo., Japan), a precolumn (10 mm × 4 mm, 5 μm; ShimadzuCo.), and a PDA detector (ShimadzuCo.) were used in the workstation. The mobile phase was acetonitrile in 0.01 M KH_2_PO_4_ solution (7:93 in volume), the flow rate was 0.5 ml min^-1^, the column temperature was 30°C, and the detection wavelength was 236 nm. A series of plasma samples with gradient concentration of vancomycin (100, 50, 25, 12.5, 6.25, 3.125, and 1.5625 μg ml^-1^) was prepared with vancomycin standard and blank plasma. After using the same parallel method for sample preparation as mentioned above, the standard samples were simultaneously injected with the samples for testing. The standard calibration curve was plotted with the peak areas and plasma concentrations of the vancomycin standard.

### Population Pharmacokinetic Modeling

Data processing and pharmacokinetic analysis were carried out with the nonlinear mixed-effects model program (NONMEM^®^, Version 7.4; Icon Inc, PA, USA), compiled with GFortran (Version 4.9.2; http://www.gfortran.org). The output was explored by the R package (Version 3.3.1; http://www.r-project.org) and Xpose (Version 4.5.3; http://xpose.sourceforge.net). The first-order conditional estimation method with η-ε interaction (FOCE-I) was used throughout the model-building procedure.

Based on the data characteristics, as vancomycin is an inabsorbable drug and was administered intravenously, and only C_min_ was available, the concentrations were analyzed according to a one-compartment pharmacokinetic model. Parameters of the structural model that were to be estimated were vancomycin clearance (CL) and volume of distribution (V).

First, we established the base compartment model. For the statistical modeling, exponential model was used as the inter-individual variation model:

$$P_{\text{ij}}=\text{TV}(P_{j})\times e^{\eta \text{ij}}$$

In this formula, P_ij_ is the pharmacokinetic parameter value of a subject, and TV(P_j_) is the typical population value of the parameter, and η_ij_ is an individual variation with normal distribution, and its mean is 0 and variance is ω^2^.

For the residual variation modeling, additive, proportional, and mixed models were applied. The additive and proportional mixed model is as follows:

$$Y_{\text{ij}}=F_{\text{ij}}\times (1+\varepsilon_{1\text{ij}})+\varepsilon_{2\text{ij}}$$

In this formula, Y_ij_ is the observed value, F_ij_ is the model prediction, and ε_1ij_ and ε_2ij_ are the residual variations whose means are 0 and variances are $\sigma_{1}^{2}$ and $\sigma_{2}^{2}$, respectively.

The modeling process included the following steps: (a) establishment of the residual model; (b) examination of the stability of the model by changing the initial value to obtain the global minimum value.

The assessment and screening of covariates for compartment modeling process were carried out by a step-by-step method to keep only the covariates with the largest contribution to predict vancomycin pharmacokinetics in a final multivariate model. In the forward model building step, the covariates were assessed for their suitability depending on the changes in the objective function values (OFV) and the inter-individual variations and residual variations. Taking an example with df = 1, when the decrease of OFV is more than 3.84 (P < 0.05, df = 1), the introduced factor can be considered as a significant covariate for the parameter model. During the backward analysis, a covariate was removed from the model every time, except when the OFV value increased by more than 6.63 units (P < 0.01, df = 1).

For the effects of continuous variables on pharmacokinetic parameters, linear, exponential, and power function models were used. The linear function model was:

P=TV(P)×(1+θ×covariate/reference)

For the influence of categorical variables, the method of assigning each variable and introducing it into model was adopted with power function models:

$$P=\text{TV}(P)\times {\left(\frac{\text{covariate}}{\text{reference}}\right)}^{\theta }$$

In these formulas, P is the individual value of the parameter, TV(P) is the typical value of the parameter, and θ is the coefficient to be computed (representing the contribution of different covariates to the pharmacokinetic parameters).

The overall diagnostic evaluation of the basic model and final model was also performed by using goodness-of-fit plots model evaluation method.

### Model Application

The final population model was used to obtain dosing regimens of vancomycin in order to reach AUC_0-24h_/MIC≥400 which is known as effective therapeutic outcome. When MIC = 1 mg L^-1^, the daily dose can be calculated by the final model of CL (L h^-1^) and the following equation:

DOSE(mg/day)=400×CL

Regimen design was performed in virtual patients with different renal function and weight according to previous reports and guideline with specific revisions (Adane et al., 2015; Lin et al., 2016; Ji et al., 2018), to determine the most appropriate dosage to meet the therapeutic criteria. The daily dose needed for patient can be calculated by extrapolating the patient’s pharmacokinetic parameter CL from our population pharmacokinetic model. Since WT and GFR are the main factors that influence CL (as shown below), and we combined the practical convenience for clinical dosage adjustment, WT was allocated into 40, 50, and 60 kg according to the common weight of Chinese kidney transplant patients. Similarly, according to standards for CKD stages, the GFR was divided into five segments, 30, 45, 60, 75, 90 ml min^-1^. Monte Carlo simulations were performed 1,000 times for different dosing scenarios to simulate and calculate drug concentration and AUC. If 90% of patients achieved the treatment goal (AUC_0-24h_/MIC≥400), the dose was considered to be an effective daily dose. All the cases were in accordance with kidney transplant recipients enrolled in this research.

## Results

### Data Collection and Vancomycin Concentration Monitoring

Data for the analyses were collected from 56 kidney transplant recipients (as shown in **Table 1**). The data and patient characteristics were distributed into five categories, and except for a few, all data are expressed as mean ± standard deviation (SD) or medians and interquartile ranges, and ranges have been also provided in case of skewed distribution. All the 56 recipients (35 males and 21 females) received renal grafts from the brain-dead donors. The estimated GFRs (and creatinine clearance/CL-Cr, not shown) were extrapolated from the sCr data. The administration-related information for concomitantly used immunosuppressants is also provided in **Table 1**.

**Table 1:** Characteristics and parameters of the included kidney transplant patients.

	Mean	SD	Median	Interquartile range	Range
***Basal Feature***					
No. of transplant recipients (n, male/female)	56 (35M/21F)				
Age of patients (years)	43.72	9.92	43.5	36–51.25	24–70
Body weight (kg)	58.27	8.47	59.95	52.2–64.52	37.7–79
Dialysis duration pretransplantation (months)	53.46	26.49	48	36–72	7–120
***Laboratory Examination***					
Aspartate aminotransferase (AST) (IU L^-1^)	18.11	10.39	16	13–19	7–75
Alanine aminotransferase (ALT) (IU L^-1^)	20.34	17.5	14.5	10–24.25	3–106
Proteinaemia (PROT) (g L^-1^)	60.08	6.48	59.15	55.42–63.92	49.4–77.5
Haemoglobin content (g L^-1^)	9.85	1.31	9.62	8.99–10.62	7.66–13.5
Serum creatinine concentration (μM)	253.98	252.63	164	141–283	60–1,490
Glomerular filtration rate (GFR) (ml min^-1^)	41.95	25.46	39.91	32.53–59.44	3.38–108.61
***Surgery Parameter***					
Cold ischaemic duration (min)	844.2	655	675	274.75–1,286	15–2,380
Kidney donors (n, brain-dead/living)	56(56/0)				
***Medical Care***					
Hospitalization duration preoperation (days)	8.2	2.05	8	7–9	4–16
Vancomycin treatment duration postoperation (days)	12.1	6.58	9	8–14	7–45
First monitoring time postoperation (day)	3.9	2.07	4	3–5	2–11
Last monitoring time postoperation (day)	8.3	2.04	8	7–9	4–16
***Immunosuppresive Intervention***					
Prednisone (55, qd) (mg d^-1^)	17	4.47	20	15–20	5–20
Mycophenolate mofetil (55, bid) (mg d^-1^)	1,281.09	296.13	1,440	1,080–1,500	500–2,000
Cyclosporine A (4, bid) (mg d^-1^)	400	282.8	300	200–500	200–800
Tacrolimus (52, bid) (mg d^-1^)	5.84	2.27	6	4–8	0.5–10
No. of patients with additional 0.5 mg tacrolimus (irreg/qd)	30(29/1)				

qd, once a day; bid, twice a day; irreg, irregular (when needed); SD, standard deviation.

The initial doses were administered once daily, and the following doses were administered 1–4 times daily. All the dosages the data on concentrations were quite divergent.

**Figure 1:**
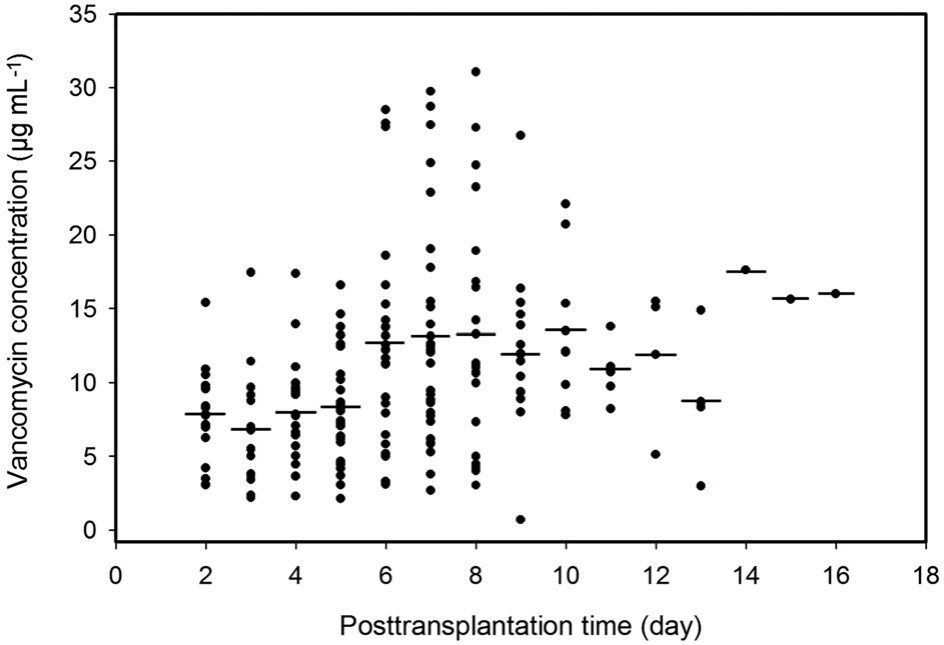
Plasma trough concentrations of vancomycin over time postoperation. Solid dots, observed points of vancomycin concentration; short lines, averages of plasma vancomycin concentrations in the same postoperation day.

The C_min_ points of vancomycin of the 56 patients during the postoperative period are shown in **Figure 1**, which include the 195 plasma C_min_ available for population modeling and indicate wide interpatient variability in the handling of vancomycin in this renal transplant patient group.

### Distribution and Correlation Analysis of Covariates

**Figure 2:**
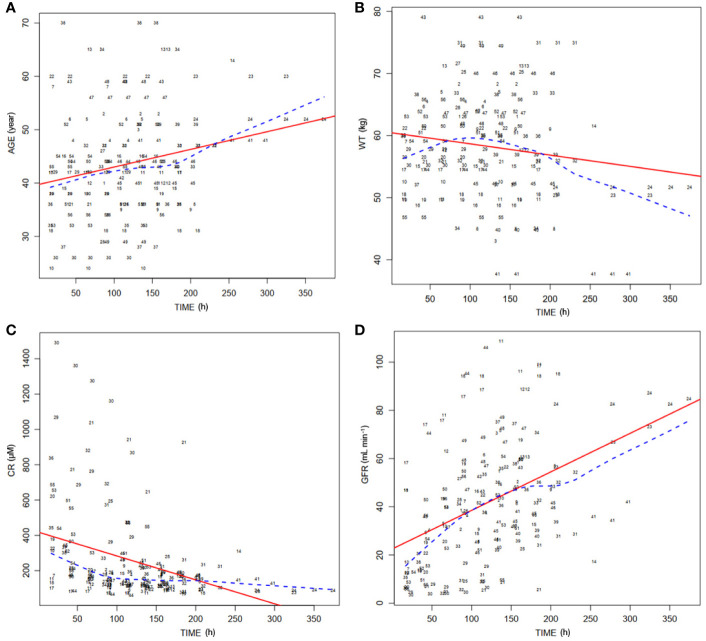
Distribution of covariates of 56 patients over time posttransplantation. **(A)** Distribution of ages of patients (AGE) in the postoperation duration. Dashed line, medians of the ages; solid line, fitting curve of ages over time. **(B)** Distribution of body weights of patients (WT) during posttransplantation period. Dashed line, medians of WT; solid line, fitting curve of WT over time. **(C)** Distribution of serum concentrations of creatinine (CR) in the postoperation duration. Dashed line, medians of CR; solid line, fitting curve of CR over time. **(D)** Distribution of theoretical glomerular filtration rates (GFR) during posttransplantation period. Dashed line, medians of GFR; solid line, fitting curve of GFR over time.

Distributions of covariates during postoperative period were investigated. Distribution of the important covariates (age, WT, sCr, and GFR) during the time following transplantation are shown in **Figures 2A–D**, respectively.

### Assessment of Covariates and Evaluation of Models

We compared three types of residual modeling: additive, proportional, and mixed models. Compared with residual models, the proportional modeling method had the lowest OFV value and better estimation precision.

A stepwise method was used to screen the covariates that affected the pharmacokinetic parameters CL and V and to determine the final model of the parameters. In the forward inclusion and backward exclusion processes, categorical variables such as combined medications including imipenem, and continuous variables such as endogenous creatinine concentration and age, were excluded. Only GFR (ml min^-1^) and WT (kg) were retained in the final formulas for CL and WT (kg) for V. Results of population pharmacokinetic analysis and final model parameters are summarized in **Table 2**. The respective final relationships describing CL (L h^-1^) and V (L) are:

$$\text{CL}=2.08\times [{\left(\text{WT}/59.95\right)}^{1.07}]\times [{\left(\text{GFR}/36.67\right)}^{0.698}]$$

$$V=63.2\times [{\left(\text{WT}/59.95\right)}^{0.934}]$$

**Table 2:** Population pharmacokinetic parameters and results of base model and the final model.

Parameter	Description	Value	CV
Base model			
OFV	Objective function value	726.347	
CL	Typical value of CL (L h^-1^)	1.84	7.6%
V	Typical value of V (L)	45.5	5.9%
ω1	Intersubject variance of CL	53.2%	19.5%
σ1	Residual proportional variance of vancomycin concentration	26.6%	20.7%
Final model			
OFV	Objective function value	596.624	
CL	Typical value of CL (L h^-1^)	2.08	3.4%
V	Typical value of V (L)	63.2	6.7%
θ1	Influential factor for GFR on CL	0.698	7.7%
θ2	Influential factor for WT on CL	1.07	20.3%
θ3	Influential factor for WT on V	0.934	43.1%
ω1	Intersubject variance of CL	21.5%	33.7%
σ1	Residual proportional variance of vancomycin concentration	24.2%	20.2%

WT, body weight; CV, coefficient of variation (relative standard deviation).

**Figure 3:**
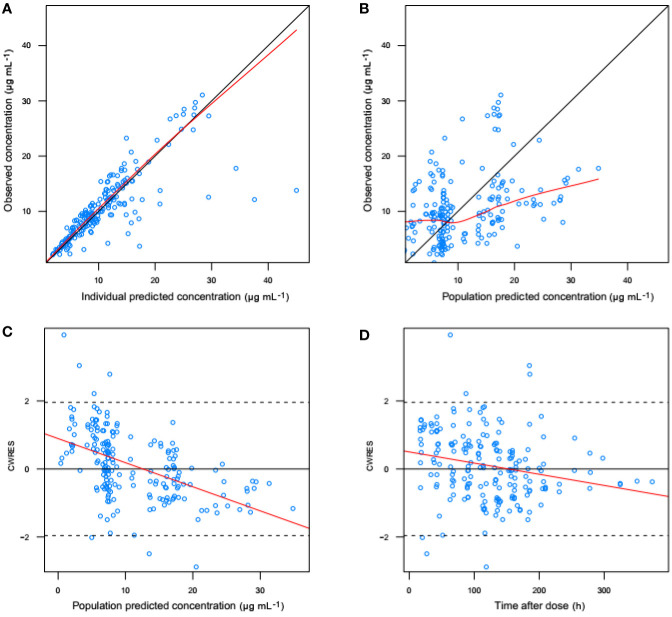
Goodness-of-fit evaluation plots obtained for the basic model. **(A)** Observed trough plasma concentrations of vancomycin versus individual model predictions of concentrations. Solid line, the line of identity. **(B)** Observed trough plasma concentrations of vancomycin versus population model predictions of concentrations. Solid line, the line of identity. **(C)** Conditional weighted residuals (CWRES) versus population predictions of concentration. **(D)** Conditional weighted residuals versus time after the initial administration of vancomycin posttransplantation.

**Figure 4:**
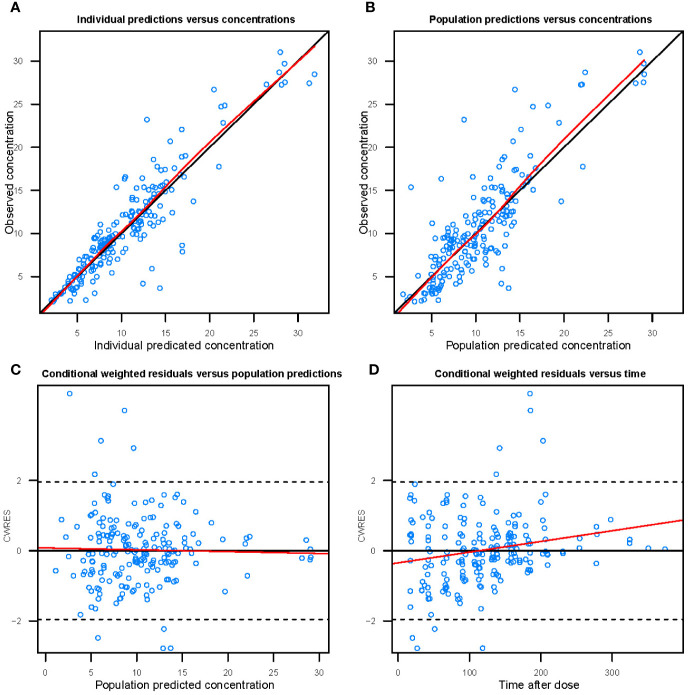
Goodness-of-fit evaluation plots of the final model. **(A)** Observed trough plasma concentrations of vancomycin versus individual model predictions of concentrations. Solid line, the line of identity. **(B)** Observed trough plasma concentrations of vancomycin versus population model predictions of concentrations. Solid line, the line of identity. **(C)** Conditional weighted residuals (CWRES) versus population predictions of concentration. **(D)** Conditional weighted residuals versus time after the initial administration of vancomycin during posttransplantation period.

**Figure 3** shows the goodness-of-fit plots obtained for the base model. **Figure 4** depicts the goodness-of-fit plots obtained for the final model. Compared with the base model, the final model showed no obvious bias or significant trends within these scatterplots. Moreover, the data fitting for the final model was much improved relative to that of the base model.

**Figure 5:**
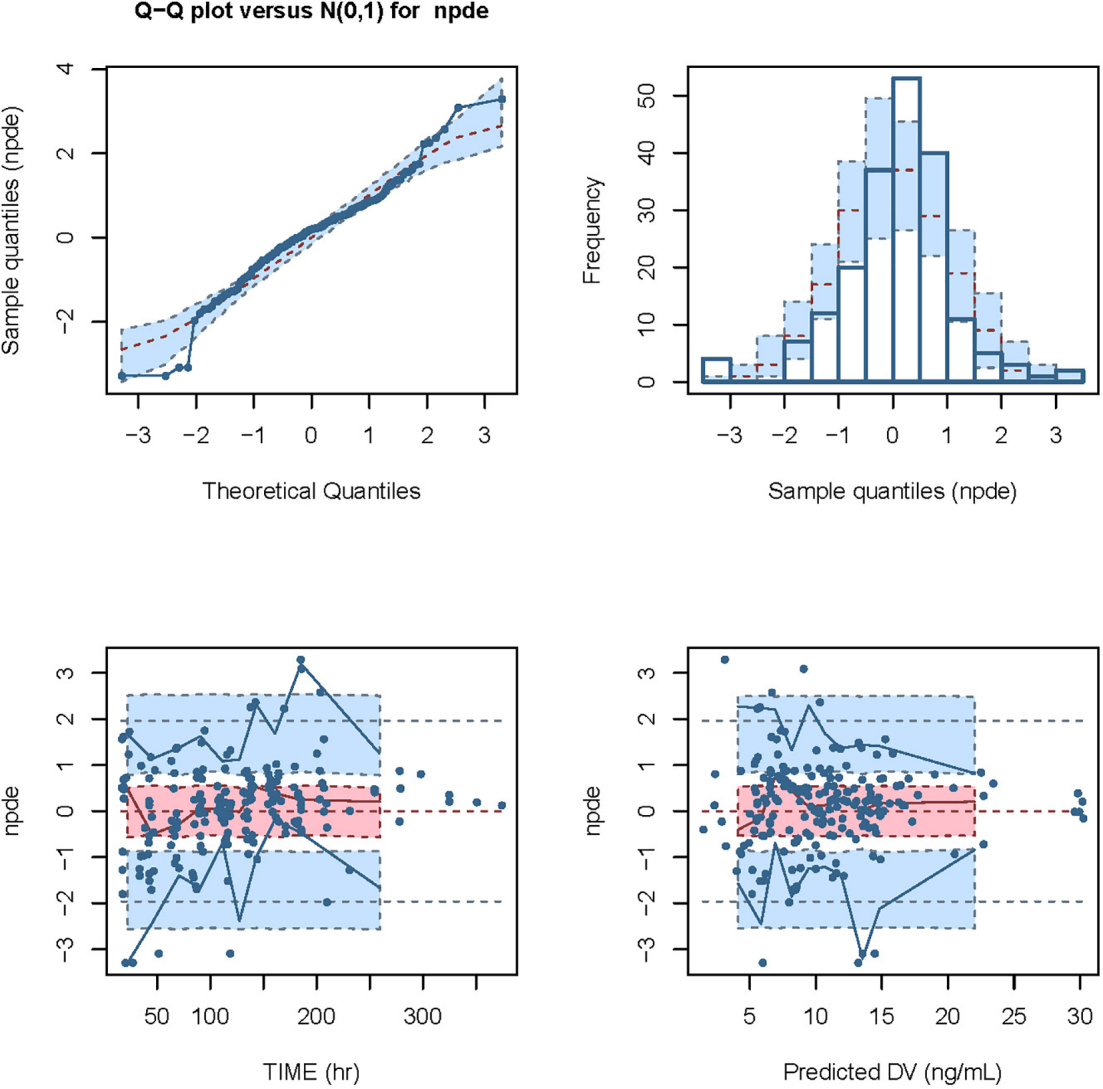
Modeling diagnosis and evaluation with NPDE analysis. The mean bias (± SE) of normalized prediction distribution errors (NPDE) was 0.09309 (± 0.071), variance (± SE) was 0.9898 (± 0.1), skewness was -0.3718, and kurtosis was 2.013. The statistical values of t-test and Fisher variance test were 0.193 and 0.947, respectively. The result of Shapiro-Wilk (SW) test for normality was 2.49 × 10^-5^, and the global adjusted p-value was 7.48 × 10^-5.^

We conducted modeling diagnosis and evaluation with normalized prediction distribution errors (NPDE) analysis method. NPDE distribution plots and the characteristic values including the mean bias of NPDE, variance, skewness, and kurtosis are shown in **Figure 5**. Statistical values of t-test and the Fisher’s variance test were 0.193 and 0.947, respectively. Shapiro-Wilk normality (SW) test value (W) and global adjusted p-value were both less than 0.001.

### Dosage Design

The dosages recommended through this model for virtual patients are displayed in **Table 3**. The recommended dosage scheme is designed based on previous reports and guideline with specific modifications (Adane et al., 2015; Lin et al., 2016; Ji et al., 2018), and is to be used in kidney transplant recipients with different segmented renal functions and body weights, and can also be adjusted according to clinical practice. The percentages of AUC_0-24h_/MIC≥400 were calculated, the scheme presents that 90% patients in different scenarios could meet the target.

**Table 3:** Final administration strategy according to PPK model.

Weight (kg)	GFR (ml/min)	Scheme:Daily dose(mg)	Percentage*of AUC_0-24h_≥400
40	30	750	>90%
	45	1,000	>90%
	60	1,250	>90%
	75	1,500	>90%
	90	1,750	>90%
50	30	1,000	>90%
	45	1,250	>90%
	60	1,500	>90%
	75	1,750	>90%
	90	2,000	>90%
60	30	1,250	>90%
	45	1,500	>90%
	60	1,750	>90%
	75	2,250	>90%
	90	2,500	>90%

The percentage of AUC_0-24h_ ≥ 400 in 1,000 times Monte Carlo simulation.

## Discussion

In this study, we analyzed the pharmacokinetic parameters of vancomycin in the adult kidney transplant recipients and identified the factors influencing its pharmacokinetics by a population pharmacokinetic approach. A one-compartment open model was optimal and adopted for the modeling of the data. Among the collected variables, only WT and GFR were found to be the significant covariates of the pharmacokinetic parameters CL and V of intravenous vancomycin.

For the current analysis, a typical value of vancomycin CL was 2.08 L h^-1^, and a typical value of vancomycin V was 63.2 L. Our study is the first retrospective analysis specifically addressing vancomycin pharmacokinetics in kidney transplant recipients after transplantation surgery, the clearance of vancomycin in our study is 2.08 L/h, with the median value of GFR is 39.91 ml/min, renal function is positive correlation with vancomycin clearance, and the findings support the previous studies on the pharmacokinetics of vancomycin. Vancomycin clearance is 8.52 L/h in patients with augmented renal clearance, and clearance is 7.56 L/h in adult Chinese patients with post-craniotomy meningitis and typical creatinine clearance rate in this study is 104.7 ml/min (Adane et al., 2015; Lin et al., 2016), in two other studies, the typical creatinine clearance rate is nearly 60 ml/min that with typical value of drug clearance is 3.35 and 2.45 L/h, respectively (Ji et al., 2018; Lin et al., 2019). Thus, drug clearance in this study might be lower than other previous Chinese studies.

WT was identified as a major covariate describing the change in CL of vancomycin in the kidney transplant recipients in postoperative period. WT and body mass index (BMI) have been identified as a risk factor for potentially suboptimal serum concentration of vancomycin in patients (Crass et al., 2018; Moffett et al., 2019; Smit et al., 2019). It has also been reported that the CL and V of vancomycin are related to WT and body sizes (Pai et al., 2017; Dunn et al., 2019; Pokorná et al., 2019). The apparent distribution volume is related positively to WT, considering equal body fat percentage. Patients with a higher WT may have lower drug concentrations for the same doses because of their commonly larger V and CL.

The estimated GFR was another important covariate that influenced CL of vancomycin in the kidney transplant recipients. GFR has been already identified as a major covariate for the pharmacokinetic parameters CL of vancomycin in patients with infectious diseases (Kovacevic et al., 2020). CL of vancomycin was lower in patients with decreased creatinine clearance due to aging, and the vancomycin CL was higher in patients with augmented GFR (Usman et al., 2018; Molina et al., 2019). Other studies in adults have reported that increased daily doses of vancomycin were required to maintain similar levels of vancomycin C_min_ in surgical and thermal injury patients due to augmented GFR and vancomycin CL (Lin Wu et al., 2015; Elder et al., 2018).

There are some limitations to our study. The main limitation of this study is the small sample size of our data set. Data from only 56 patients (195 plasma C_min_) were applied to establish the model. Therefore, the results should be generalized carefully, and further evaluation studies should be conducted when more samples are collected. In addition, Renal function in renal transplant patients is unstable. Under these conditions, GRF cannot be estimated accurately and it is difficult to interpret a measured concentration as it cannot represent steady state in an unstable patient. It is not accurate to estimate GFR in renal function recovery stage of renal transplant patients by EPI, but there is no better model to estimate the renal function of patients at this stage. This model estimation can roughly reflect the recovery of renal function of patients in the actual work. The application of this estimation value to guide the adjustment of drug dosage can also help to reduce the safety problems of vancomycin.

Additionally, we have used the population pharmacokinetic model to develop clinical dosing strategies in case the vancomycin concentration cannot be detected in a timely manner. Using this dose adjustment strategy, we can design the dosage of vancomycin in advance for the renal transplant patients once creatinine value is available to achieve better treatment goals and reduce adverse drug reactions.

## Conclusion

In summary the relative importance of factors influencing vancomycin pharmacokinetic parameters and disposition in adult kidney transplant recipients was assessed, and WT and GFR were identified as significant covariates for CL and V. In clinical renal transplant patients, WT and GFR should be considered individually for vancomycin therapy according to recommended dosage scheme to avoid insufficient exposure-induced ineffective treatment and resistance and overdosing-induced toxicities.

## Data Availability Statement

All datasets presented in this study are included in the article/supplementary material.

## Ethics Statement

The studies involving human participants were reviewed and approved by The First Affiliated Hospital, Zhejiang University. The patients/participants provided their written informed consent to participate in this study.

## Author Contributions

K-fM collected the data and wrote the manuscript. PY and J-yW collected the data. Y-xL, ZJ, and J-hL analyzed the data. SY oversaw the project, wrote, and revised the manuscript. All authors contributed to the article and approved the submitted version.

## Funding

The project was supported by the Natural Science Foundation of Zhejiang Province (No. LY19H310008, LQ18H310001) and Medical Science and Technology Project of Zhejiang Province (No. 2018RC031, 2017KY339).

## Conflict of Interest

The authors declare that the research was conducted in the absence of any commercial or financial relationships that could be construed as a potential conflict of interest.
